# Predictors of Self-Rated Oral Health Among Community-Dwelling Adults With Diabetes: A Cross-Sectional Analysis

**DOI:** 10.7759/cureus.97454

**Published:** 2025-11-21

**Authors:** Ya-Ching Huang, Rosaleen D Bloom, Subash Sapkota, Alfiya Shaikh Mohd Rafiq, Root Bahlbi, Valerie Zapien

**Affiliations:** 1 College of Nursing, Texas A&M University Health Science Center, Bryan, USA; 2 School of Public Health, Texas A&M University Health Science Center, College Station, USA; 3 College of Education, Texas State University, San Marcos, USA

**Keywords:** diabetes, oral health, self-management, self-rated health, self-rated oral health

## Abstract

Background

This study aims to identify predictors of self-rated oral health (SROH) among community-dwelling adults with diabetes. Given that SROH has been associated with regular dental visits, oral health conditions, and emotional well-being, understanding its predictors may help identify individuals at higher risk for poor oral health outcomes and develop targeted interventions.

Methodology

This study conducted a secondary data analysis of two cross-sectional surveys among adults with diabetes. In addition to descriptive analysis, a logistic model of SROH was estimated with the following set of predictors: demographics (age, ethnicity, gender, marital status, income, health insurance, and dental insurance), health condition (number of chronic diseases and hemoglobin A1c level), and self-rated health (SRH).

Results

There were 136 participants in this study, with a mean age of 58.94 ± 13.36 years. Overall, 52 (38%) were white, 80 (58.8%) were female, 80 (58.8%) had a high school or higher degree, and 91 (67%) rated their oral health as good. In the logistic regression model of SROH, family income that meets needs (odds ratio (OR) = 0.34, 95% confidence interval (CI) = 0.13, 0.90) and the presence of dental insurance (OR = 0.35, 95% CI = 0.14, 0.89) were identified as protective factors against poor SROH. Furthermore, individuals who reported poor SRH were at greater odds of reporting poor SROH (OR = 2.61, 95% CI = 1.02, 6.64).

Conclusions

The findings from this study provide information on significant predictors of SROH in individuals with diabetes. This knowledge helps identify high-risk individuals and introduce tailored interventions to increase the quality of dental care and improve oral health and overall well-being.

## Introduction

Oral health is increasingly recognized as an integral component of general health. Evidence suggests multiple systemic conditions, including, but not limited to, pulmonary disease, osteoporosis, kidney disease, diabetes, and pregnancy-related complications, are associated with periodontal disease. However, research is still needed to determine if this association is causal or coincidental [1]. While the American Diabetes Association (ADA) recommends that individuals with diabetes schedule at least two dental check-ups each year, real-world adherence falls significantly short of this target [2]. Specifically, in the United States, 65.5% of adults reported having a dental visit in 2019 [3]. Compounding this issue, Wei et al. reported that approximately 60% of U.S. adults with diabetes had a medical visit within the past year but did not receive any dental care during the same period. Furthermore, adults aged 20 years and older with diabetes were found to be 40% more likely to have untreated dental cavities compared to people without diabetes [4].

Having fewer than two dental visits per year not only impacts oral health but also predisposes patients to other systemic illnesses [5]. Regarding periodontal disease, a bidirectional relationship between diabetes and periodontal disease is well established, where patients with diabetes are at increased risk for periodontal disease, and those with active periodontal disease are at increased risk for poor glycemic control [6]. High blood glucose levels impair the body’s defense mechanism by weakening white blood cells and thereby increasing susceptibility to oral infections. Hyperglycemia also leads to increased glucose levels in saliva. This excess glucose is a good nutrient source for bacteria present in dental plaque. The net effect is an increased risk of cavities and gum disease [7]. Conversely, chronic inflammation from infections caused by cavities and periodontal disease increases stress in the body, leading to higher blood glucose levels, which, in turn, leads to further inflammation [8]. Most common oral complications associated with diabetes include oral candidiasis, dry mouth, tooth loss, halitosis, burning mouth syndrome, geographical tongue, dental caries, and periodontal disease [9]. Given this evidence, regular dental visits for patients with diabetes are essential.

One of the proxy measures utilized to assess frequency of dental visits, oral health, self-esteem, and life satisfaction is self-rated oral health (SROH) [10-12]. At a population level, it is not always possible to evaluate oral health status by direct clinical examination done by a healthcare provider; therefore, indirect predictors of oral health status, such as SROH, can be utilized in such cases and are more cost-effective. Although studies focusing on predictors of SROH in the general population are widespread [13-15], studies focusing on predictors of SROH in people with diabetes are limited. Therefore, this study aims to identify the predictors of SROH among 136 community-dwelling adults with diabetes.

## Materials and methods

Study design and data collection

This study utilized secondary data from two independent, cross-sectional studies. The first study examined oral health knowledge and the risk of periodontitis among 93 adults with type 2 diabetes from community settings. Data were collected between March 2020 and June 2022, during the principal investigator’s (PI) tenure at Texas State University. The second study was part of a community-based health promotion initiative and included 43 adults with type 1 or type 2 diabetes. Data collection for this study occurred between July 2023 and December 2023, during the PI’s current appointment at Texas A&M University. Data from both parent studies have not yet been published. Ethical approval for each study was obtained from the respective institutional review boards (approval numbers: 7019 and IRB2023-0345D). All participants provided written informed consent after being informed that their deidentified, aggregated data might be used in future publications or reports.

The convenience sampling method was utilized in both studies. Participants were recruited through flyers and presentations at community-centered events, including health clinics, churches, farmers’ markets, and health fairs, with a focus on reaching community-dwelling populations. Eligibility criteria included being 18 years of age or older, self-reporting a diagnosis of diabetes (type 1 or type 2), and the ability to read or communicate in either English or Spanish. Individuals were excluded from the study if they had a diagnosis of gestational diabetes or a known cognitive impairment that could interfere with their ability to provide informed consent or accurately respond to survey questions. The datasets were combined due to shared variables and similar survey structures relevant to the current study objectives.

Measures

Demographics

Participants reported their age (in years), race (coded as 0 for non-Hispanic white, 1 for Hispanic, 2 for Black, and 3 for others), sex (coded as 0 for male and 1 for female), marital status (coded as 0 for single and 1 for married or living with a significant other), financial status (coded as 0 for income meeting family needs and 1 for income not meeting family needs), level of educational attainment (0 for <12 years education and 1 for ≥12 years education), health insurance status (coded as 0 for absence of health insurance and 1 for presence of health insurance), and dental insurance status (coded as 0 for absence and 1 for presence of dental insurance).

Physical Health Factors

Data on hemoglobin A1c (HbA1c) level and comorbidities were collected. Assessment of HbA1c was performed using the A1CNOW®+ point-of-care kits (PTS Diagnostics, Whitestown, Indiana) operated by the research team members and trained pre-licensure nursing students who were supervised by the research team. The A1CNOW® is an FDA-approved device for home and professional use and has demonstrated consistency and accuracy with clinical laboratory results [16] and has been used in other diabetes studies [17]. The number of comorbidities was measured with a nine-item list of chronic diseases other than diabetes, including hypertension, heart disease, stroke, cancer, arthritis, hepatitis, kidney disease, asthma, and chronic obstructive pulmonary disease. Participants reported their conditions, and their responses were summed up to create a score for analysis. A higher score indicated a greater number of comorbidities.

Self-Rated Health and Self-Rated Oral Health

Self-rated health (SRH) and SROH were measured by asking the following questions: “How would you rate your overall health at the present time?” and “Overall, how would you rate the health of your teeth and gums?” There were five Likert response choices (0 = excellent, 1 = very good, 2 = good, 3 = fair, and 4 = poor), with lower scores indicating better SRH and SROH. For logistical regression analysis, we created dichotomous variables by grouping 0 to 2 as “good” for SRH and SROH (coded as 0), and scores 3 and 4 as “fair/poor” (coded as 1).

Data analysis

SPSS Version 29 for Windows (IBM Corp., Armonk, NY, USA) statistical software was used for data management and analysis. Descriptive statistics (means, standard deviations, frequency distributions, and percentages) were calculated for all variables. Independent t-test and chi-square test were employed to compare the differences in demographic, physical factors, and SRH between the good SROH and fair/poor SROH groups. A multivariable logistic regression was conducted to understand the independent effects of demographics, physical factors, and SRH on SROH. Statistical significance was set at p-values <0.05. The overall fit of the logistic regression model was assessed using the Omnibus test of model coefficients, which was statistically significant (χ^2^ = 25.15, p = 0.02), indicating that the model provides a better fit to the data than a model with no predictors.

## Results

Table 1 shows the participants’ characteristics and the descriptive analysis results of this study. In total, 136 participants completed the survey, with a mean age of 58.94 ± 13.36 years. Most participants were non-Hispanic whites (38.2%), followed by Hispanics (36.8%) and Blacks (14.7%). The sample population was predominantly female (58.8%). Most participants were married (57.4%), and the majority (73.5%) had the necessary family income to sustain daily needs. Around 59% of the participants had a high school degree or more. Although 84.6% of the participants had medical health insurance, only 59.6% had dental insurance.

**Table 1:** Descriptive characteristics of the study sample (N = 136). A chi-square test was used to examine the association between SROH and categorical variables (demographic factors and SRH). Similarly, an independent-sample t-test was used to compare the mean (number of other chronic diseases, HbA1C level) between two SROH groups. SROH = self-rated oral health; SRH = self-rated health; HbA1c = hemoglobin A1c

	Total (n = 136)	Good SROH (n = 91)	Poor SROH (n = 45)	P-value
	n (%) or mean ± SD	n (%) or mean ± SD	n (%) or mean ± SD	
Demographic factors				
Race				0.383
Non-Hispanic white	52 (38.2%)	39 (42.9%)	13 (28.9%)	
Hispanic	50 (36.8%)	32 (35.2%)	18 (40%)	
Black	20 (14.7%)	11 (12.1%)	9 (20%)	
Other	14 (10.3%)	9 (9.9%)	5 (11.1%)	
Sex				0.862
Male	56 (41.2%)	37 (40.7%)	19 (42.2%)	
Female	80 (58.8%)	54 (59.3%)	26 (57.8%)	
Age	58.94 ± 13.36	59.07 ± 12.98	58.68 ± 14.23	0.874
Marital status				0.076
Married	78 (57.4%)	57 (62.6%)	21 (46.7%)	
Single	58 (42.6%)	34 (37.4%)	24 (53.3%)	
Family income meeting needs				0.008
Yes	100 (74.1%)	73 (81.1%)	27 (60%)	
No	35 (25.9 %)	17 (18.9%)	18 (40%)	
High school degree or above				0.017
Yes	80 (58.86%)	60 (65.93%)	20 (44.44%)	
No	56 (41.17%)	31 (34.06%)	25 (55.55%)	
Have health insurance				0.301
Yes	115 (84.6%)	79 (86.8%)	36 (80%)	
No	21 (15.4%)	12 (13.2%)	9 (20%)	
Have dental insurance				0.012
Yes	81 (59.6%)	61 (67%)	20 (44.4%)	
No	55 (40.4%)	30 (33%)	25 (55.6%)	
Physical health factors				
Number of other chronic diseases	2.19 ± 1.43	2.16 ± 1.38	2.24 ± 1.55	0.762
HbA1c level (%)	6.74 ± 1.48	6.79 ± 1.60	6.65 ± 1.21	0.612
SRH				0.031
Good SRH	103 (75.7%)	74 (81.3%)	29 (64.4%)	
Fair/Poor SRH	33 (24.3%)	17 (18.7%)	16 (35.6%)	

A descriptive analysis was conducted to study the distribution of physical health factors among participants. Omitting diabetes, the mean number of other chronic diseases among participants was 2.19 ± 1.43. Furthermore, the mean HbA1c level was 6.74 ± 1.48%. Most participants (75.7%) reported having good SRH.

When comparing SROH groups, 91 (67%) reported good SROH, while 45 (33%) reported fair or poor SROH. The prevalence of good SROH was significantly higher among participants whose family income was sufficient to meet their needs (χ² = 6.96, p < 0.001), those with at least a high school education (χ² = 10.32, p < 0.05), those with dental insurance (χ² = 6.38, p < 0.05); and those reporting good SRH (χ² = 4.66, p < 0.05).

A multivariable logistic regression analysis was conducted to identify significant predictors of good versus fair/poor SROH (Table 2). After accounting for all other variables, poor SROH was significantly associated with insufficient financial resources, lack of dental insurance, and fair or poor SRH. Participants who reported having sufficient family income to meet daily needs had significantly lower rates of fair or poor SROH compared to those with insufficient income (odds ratio (OR) = 0.34, 95% confidence interval (CI) = 0.13, 0.90). Similarly, individuals with dental insurance had lower odds of reporting fair or poor SROH than those without insurance (OR = 0.35, 95% CI = 0.14, 0.89). Conversely, participants who rated their general health as fair or poor had significantly higher rates of reporting fair or poor SROH compared to those with good SRH (OR = 2.61, 95% CI =1.02, 6.64).

**Table 2:** Logistic regression model of SROH with the good SROH category as a reference group. *: statistically significant at p-values <0.05. SROH = self-rated oral health; SRH = self-rated health; HbA1c = hemoglobin A1c; OR = odds ratio; CI = confidence interval

	OR	95% CI	P-value
Demographic factors			
Age	0.99	(0.95, 1.03)	0.731
Race			
Non-Hispanic white	Reference		
Hispanic	2.14	(0.60, 7.68)	0.240
Black	1.52	(0.50, 4.56)	0.453
Others	3.05	(0.73, 12.68)	0.125
Sex			
Male	Reference		
Female	1.87	(0.77, 4.51)	0.163
Marital status			
Single	Reference		
Married or stay with a significant one	0.67	(0.28, 1.59)	0.364
Family income meeting needs			
No	Reference		
Yes	0.34*	(0.13, 0.90)	0.031
Educational level			
Below high school degree	Reference		
High school degree or above	0.487	(0.20, 1.15)	0.104
Have health insurance			
No	Reference		
Yes	2.27	(0.60, 8.48)	0.222
Have dental insurance			
No	Reference		
Yes	0.35*	(0.14, 0.89)	0.028
Physical health factors			
HbA1c level	0.86	(0.64, 1.16)	0.346
Number of other chronic diseases	0.96	(0.70, 1.33)	0.841
SRH			
Good	Reference		
Fair/Poor	2.61*	(1.02, 6.64)	0.043

## Discussion

This study examined the predictors of SROH among community-dwelling adults with diabetes. Overall, 33% of participants rated their oral health as fair or poor. Poor SROH was significantly associated with insufficient financial resources, lack of dental insurance, and fair or poor SRH. These findings underscore the importance of addressing social determinants of health to improve oral health outcomes among individuals with diabetes. One of the contrasting features revealed by the descriptive analysis of our study population was the distribution of health insurance and dental insurance. Although most study participants (84.6%) had health insurance, only 59.6% had dental insurance. This contrast is also prevalent in the national population. In a study utilizing the Medical Expenditure Panel Survey, a dataset from 1996 to 2015, around 72% of adults (aged 21-64 years) and only 38% of older adults (65 years and above) had dental insurance coverage [18]. Many working adults lose their dental benefits following retirement, as traditional Medicare does not provide coverage for routine dental care [19].

Although race was not a statistically significant factor in distinguishing between good versus fair/poor SROH groups in this study, notable racial patterns emerged. Among participants with good SROH, the most reported race was non-Hispanic white (42.9%). In contrast, most participants in the fair/poor SROH category identified as Hispanic (40%), followed by non-Hispanic white (29%). These trends are consistent with findings reported by Wu et al. (2011), who conducted a secondary data analysis of 4,859 community-dwelling adults aged 60 years and older. Their study found that poor SROH was more frequently reported among Black (36.8%) and Hispanic (35%) participants compared to white participants (21.4%), with these racial differences being statistically significant [20]. This finding might imply the need for culturally and linguistically tailored oral health interventions.

Although education was not a significant predictor of SROH in our logistic regression model, in the descriptive analysis, participants in the good SROH category had significantly higher education levels than those in the fair or poor SROH category, showing that only 44% of participants in the poor SROH group had a high school education compared to 66% in the good SROH group. This is consistent with findings from a secondary data analysis by Li et al. (2019), which showed that U.S. adults with a college degree, who were white, and those with a family poverty income ratio ≥400% were more likely to report excellent SROH [15]. Our findings align with this pattern in terms of education and income. Lower educational levels may be associated with limited health literacy, reduced access to oral health information, and less frequent engagement in preventive dental behaviors. Public health interventions aimed at improving oral health among adults with diabetes should incorporate oral health education strategies tailored to individuals with lower educational backgrounds. Enhancing oral health literacy could empower individuals to adopt healthier behaviors, seek regular dental care, and, ultimately, improve their perceived and actual oral health status.

In our logistic regression analysis, both adequate household income and the presence of dental insurance emerged as significant protective factors against poor SROH. Specifically, 60% of individuals within the fair/poor SROH category reported having sufficient family income to meet daily needs, whereas approximately 80% of those in the good SROH category reported adequate financial resources. Although the direct relationship between income and SROH has not been extensively explored in the literature, existing evidence supports a consistent association between lower income and adverse oral health outcomes. For example, Eke et al. observed that the prevalence of periodontitis was markedly higher among individuals with low income (60%) compared to those with higher income (30%), illustrating a significant oral health disparity linked to socioeconomic status [21]. Furthermore, Badewy and Aldorssri found that dental insurance coverage was associated with an increased likelihood of reporting excellent or very good oral health (marginal effect (ME) = 6.9, 95% CI = 5.4, 8.3), as well as higher rates of dental visits (ME = 11.3, 95% CI = 9.8, 12.8) within the preceding year. Their study also noted a decrease in the likelihood of avoiding certain foods due to oral health problems (ME = 3.5, 95% CI = 1.9, 5.1) and seeking dental care exclusively in emergencies (ME = -11.2, 95% CI = -12.5, -9.9), underscoring the role of insurance in promoting proactive oral health behaviors [22]. These findings also suggest that financial resources play a critical role in perceived oral health status among adults with diabetes. Insufficient income may limit access to preventive dental care, oral hygiene products, and healthy foods, which are essential to maintaining good oral health [23]. Addressing financial barriers and expanding access to affordable dental services should be key components of public health strategies aimed at reducing oral health disparities in this population. All public health indigent healthcare plans should include dental insurance coverage for low-income people and those with diabetes and/or other systemic diseases [24]. The Supplemental Nutrition Assistance Program benefits could be expanded to include hygiene products (e.g., toothpaste, toothbrush, dental floss) to help close the gap in access to regular oral care [25].

The existing literature focusing on predictors of SROH specifically in diabetic patients is limited. Our logistic regression analysis showed that participants with fair/poor SRH were at increased odds of reporting fair/poor SROH, which is consistent with a national survey that included a sample of 37,904 U.S. adults, indicating excellent SRH serves as an independent predictor of excellent SROH [15]. These results suggest that perceived general health status may be a meaningful correlate of oral health perceptions, particularly in populations burdened by chronic conditions such as diabetes. Beyond its statistical association, the interplay between SRH and SROH in populations with diabetes may reflect overlapping physiological and behavioral pathways. Diabetes is known to exacerbate oral health conditions such as periodontitis through mechanisms linked to systemic inflammation, impaired immune response, and poor glycemic control [6,8]. These oral complications, in turn, can negatively influence patients’ perceptions of their oral health, particularly when they interfere with daily functioning, nutrition, or quality of life. Moreover, individuals with diabetes may experience diminished self-efficacy in managing both general and oral health conditions, further contributing to lower SRH and SROH scores. As such, self-rated measures may serve not only as indirect indicators of clinical burden but also as valuable markers of psychosocial strain and health inequity within chronic disease populations.

Limitations

Some limitations of this study are worth acknowledging. Although logistic regression helps predict the likelihood of poor SROH and understand the factors that influence it, a cause-and-effect relationship cannot be established because of the cross-sectional study design. Our study focused on individuals with diabetes and measured their HbA1c levels; however, we did not collect information on the duration and medication regimen of their diabetes. This missing information limits the ability to control for these variables when estimating the influence of HbA1c level on SROH. Moreover, the mean HbA1c level in our study participants was 6.74 ± 1.48%, which indicates well-controlled diabetes. Thus, a generalization of our study findings to populations with poor glucose levels should be done with caution. Although the etiology of oral health issues such as gum disease, dry mouth, delayed healing, and infection associated with hyperglycemia is generally similar in both type 1 and type 2 diabetes, our study lacked information on diabetes type and thus could not confirm this assumption. Future studies should examine patients with type 1 and type 2 diabetes separately to further investigate potential differences. Similarly, the small sample size in this study might limit the generalizability of our findings to a broader population. Convenience sampling methods, on the other hand, can lead to selection bias. Lastly, the use of self-reporting techniques can result in various biases, including information bias and social desirability bias. In a community setting, people who are less confident in their diabetes management or who are experiencing worse health conditions may be less motivated to participate in the study.

## Conclusions

The study findings increase our understanding of the predictors of SROH among people with diabetes in the community setting. This study identified key socioeconomic and health-related factors that contribute to poorer oral health outcomes. Specifically, insufficient family income, lack of dental insurance, and perceived poorer overall health were found to substantially increase the rates of negative self-assessed oral health conditions. These findings suggest that these indicators can be effectively used as proxies to identify individuals who may be at higher risk for oral health issues. By incorporating these proxies into community health screenings, healthcare providers and community health workers can proactively identify and address the oral health needs of vulnerable populations, thereby improving overall health outcomes and reducing disparities in oral healthcare access.
